# The role of photo-electric properties of silk cocoon membrane in pupal metamorphosis: A natural solar cell

**DOI:** 10.1038/srep21915

**Published:** 2016-02-24

**Authors:** Brindan Tulachan, Shivansh Srivastava, Tejas Sanjeev Kusurkar, Niroj Kumar Sethy, Kalpana Bhargava, Sushil Kumar Singh, Deepu Philip, Alok Bajpai, Mainak Das

**Affiliations:** 1 Biological Sciences & Bioengineering, Indian Institute of Technology Kanpur, Kanpur, Uttar Pradesh, 208016, India; 2 Department of Applied Chemistry and Polymer Technology, Delhi Technological University, Shahbad Daulatpur, Main Bawana Road, Delhi, 110042, India; 3 Defense Institute of Physiology & Allied Sciences, Defense Research Development Organization, Timarpur, Lucknow Road, Delhi, 110054, India; 4 Solid State Physics Laboratory, Defense Research Development Organization, Timarpur, Lucknow Road, Delhi, 110054, India; 5 Industrial & Management Engineering, Indian Institute of Technology Kanpur, Kanpur, Uttar Pradesh, 208016, India; 6 Design Program, Indian Institute of Technology Kanpur, Kanpur, Uttar Pradesh, 208016, India; 7 Institute Psychiatrist, Indian Institute of Technology Kanpur, Kanpur, Uttar Pradesh, 208016, India

## Abstract

Silkworm metamorphosis is governed by the intrinsic and extrinsic factors. One key intrinsic factor is the temporal electrical firing of the neuro-secretory cells of the dormant pupae residing inside the silk cocoon membrane (SCM). Extrinsic factors are environmental like temperature, humidity and light. The firing pattern of the cells is a function of the environmental factors that eventually controls the pupal development. How does the nervous organization of the dormant pupae sense the environment even while enclosed inside the cocoon shell? We propose that the SCM does this by capturing the incident light and converting it to electricity in addition to translating the variation in temperature and humidity as an electrical signal. The light to electricity conversion is more pronounced with ultraviolet (UV) frequency. We discovered that a UV sensitive fluorescent quercetin derivative that is present on the SCM and pupal body surface is responsible for generating the observed photo current. Based on these results, we propose an equivalent circuit model of the SCM where an overall electrical output transfers the weather information to pupae, directing its growth. We further discuss the implication of this electrical energy conversion and its utility for consumable electricity.

Silkworm has a typical four-stage lifecycle viz., egg, larvae, pupae and adult moth^1^,^2^,^3^,^4^,^5^,^6^,^7^,^8^,^9^,^10^,^11^. An adult moth lay the eggs and upon hatching the larvae emerges. The larvae voraciously eats on the plant leaves and copiously secretes a viscous salivary fluid rich in protein, which eventually forms a selectively permeable^11^, thermoregulatory^11^,^12^,^13^, UV-protecting^10^,^14^,^15^, waterproof shell^9^,^16^ around its own body, termed as silk cocoon. Entering of the larvae in this self-enclosed chamber, mark the beginning of pupal phase or commonly called dormant or diapause phase in silkworm’s life. This phase varies from 21 days to as long as 9 months in certain species of silkworm found in the temperate regions of the world^11^. Once this self-induced dormancy is completed, an adult moth emerges out of the silk cocoon. This whole process is termed as metamorphosis.

The transformation of the pupae to an adult moth is a tightly regulated neuro-electrical event under the control of intrinsic (neuro-secretory brain cells of the pupae) and extrinsic (seasonal parameters namely temperature, relative humidity, moisture and light) factors^1^,^2^,^3^,^4^,^5^,^6^,^7^,^8^. Intrinsic control is exerted by the spontaneous periodic electrical firing of the brain cells regulating the activity of the prothoracic gland^5^,^6^,^7^,^8^. Further it has been shown that the pupal dormancy could be terminated by external electrical stimulation of the pupal brain^7^, thus mimicking the spontaneous electrical signaling of the brain cells. Neuro-electrical firing pattern of the pupal brain cells is influenced by the extrinsic seasonal factors. That is the reason why metamorphosis is a season dependent event^17^.

One obvious question is how the brain cells of the dormant pupae sense the seasonal changes? There are two possible options: one option is that pupae has light, moisture and temperature sensitive cells which remain active all throughout the dormant phase of its life; alternatively the silk cocoon, which is housing the pupae during the dormant phase has inbuilt light, humidity and temperature sensors and it conveys these signals to the dormant pupae.

Here we have explored the alternative option: silk cocoon has inbuilt light, humidity and temperature sensors.

In our earlier work, we discovered that the silk cocoon membrane has an inherent ability to sense the change in temperature and humidity of the surrounding environment^9^,^11^. Any change in temperature or humidity is translated into electrical signals in the form of current flow across the silk cocoon membrane. Maximum current flow is observed when silk cocoon membrane is exposed to high temperature and high humidity^9^,^11^. In real life this means that on a hot and humid day of rainy season, silk cocoon experiences maximum current flow across its membrane as compared to a dry sunny or dry winter day. Essentially the electrical resistance across the membrane changes with changing temperature and humidity conditions^9^. Here it is noteworthy that a temperature between 25°–28 °C and a relative humidity between 75–80% is optimal for successfully rearing silkworm and that is one of the key reason why silkworm rearing is mostly concentrated in the tropical and subtropical regions of China and India^17^. Another interesting aspect of this membrane is its electric charge storage capacity over a period of time, like a battery or a capacitor^9^. Since out of the three extrinsic parameters, both humidity and temperature influences the electrical properties of the membrane^9^, so this lead us to study the effect of the third factor viz., effect of light on the electrical properties of the silk cocoon membrane.

## Results

**Figure 1:**
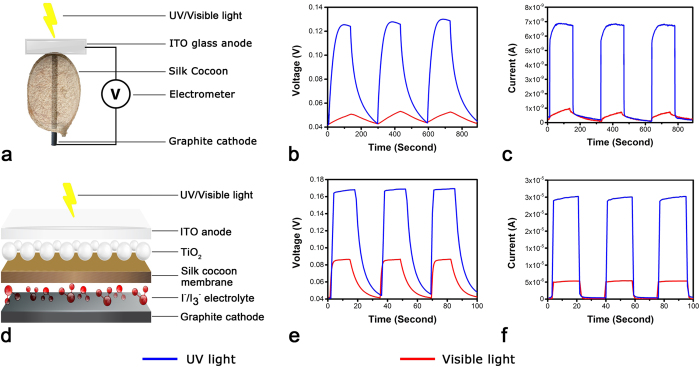
Photo-electrical properties of silk cocoon membrane. (**a**) A moist silk cocoon is placed between ITO glass served as the anode and a graphite rod passing right through the middle of the cocoon, served as the cathode. (**b**) Average voltage recordings made using the device, when the silk cocoon membrane was exposed to visible and UV light. The voltage traces illustrated here is the average value obtained from 6 different devices. Voltage recordings rose significantly upon exposure to UV light, and dropped soon after the light source is removed. (**c**) Average current recorded from the silk cocoon membrane upon exposure to visible and UV light. (**d**) Improvised silk cocoon device to measure photo-current and photo-voltage. (**e**) Voltage recording from the improvised device. (**f**) Current recording from the device.

In order to study of the effect of light on the whole silk cocoon membrane, a standard device was assembled as shown in Fig. 1a. The detail of the device is provided in the **methods section**. Simply by shining visible light onto the whole silk cocoon membrane device, we observe an average saturation voltage increase of 0.05 V and generation of an average saturation current of the magnitude of 1 × 10^**−9**^ A across the silk cocoon membrane. Interestingly when ultra-violet (UV) light is shone on to the silk cocoon surface, we observe a three fold increase in the voltage (0.125 V) and seven fold increase in current value (7 × 10^**−9**^ A) (Fig. 1b,c). Nevertheless in both the cases, response of the device was quite slow with rise and decay times longer than 2 min. Most likely, the resistance offered by the silk cocoon matrix causes the lag in response time. The silk cap covering the hornet nest in an earlier study^18^ had demonstrated a similar kind of UV dependent photocurrent.

The observed photocurrent and photo-voltage recorded from the native cocoon membrane is low (Fig. 1b,c). We made an improvised recording system to prevent the loss of voltage and current, by drawing inspiration from the design of dye sensitized solar cells^19^,^20^,^21^,^22^ (Fig. 1d). Detailed device fabrication is given in **methods section**. It is observed that current values increases by thousand fold along with an increase in the potential. The response time of the device (Fig. 1d) improved significantly as compared to the raw silk cocoon set up (Fig. 1a). In Fig. 1e,f, average voltage and current values have been plotted as a function of time. We observe an average saturation current of 5 × 10^−6^ A for visible light and 30 × 10^−6^ A for UV light. Similarly average saturation voltage of 0.083 V for visible and 0.17 V for UV light is observed. In the improvised device, we observed higher current and voltage in both the conditions. We attributed these to more efficient charge transport through nano-crystalline TiO_2_ layer and the presence of I^**−**^/I_3_^**−**^electrolyte^21^. The results confirmed that when light is shone on to the silk cocoon membrane, it has the ability to convert it into electrical signal. The most notable aspect of these two results is that photo response is markedly increased when exclusively UV light is shone on the silk cocoon surface thus hinting towards the possibility of the presence of a photo-electrically active UV sensitive component on silk cocoon surface. This characteristic raises a futuristic possibility of developing a bio-inspired UV solar cell using this surface.

In one of our earlier work we observed that the silk cocoon membrane of the wild silkworm species of *Antheraea mylitta*, commonly called Tasar silk, has acquired a fluorescent signature by virtue of the presence of a fluorescent quercetin derivative^10^,^23^,^24^. The same compound was earlier observed in domesticated silkworm species of *Bombyx mori*^14^. We showed that this fluorophore is a potential bio-imaging cum anti-oxidant agent^10^,^23^,^24^. Optical studies on this fluorophore showed a maximum absorption at a wavelength of 235 nm and a maximum emission at a wavelength of 420 nm. We have not observed any significant emission following excitations at 488 nm as well as 543 nm^10^,^23^,^24^. Our observations in previous photo-electrical studies revealed maximum photo-current upon UV exposure, so the next natural question was -‘Is this fluorophore involved in generating UV induced photo-current?’ This fluorophore otherwise is believed to act as an UV protecting shield for the developing pupae.

**Figure 2:**
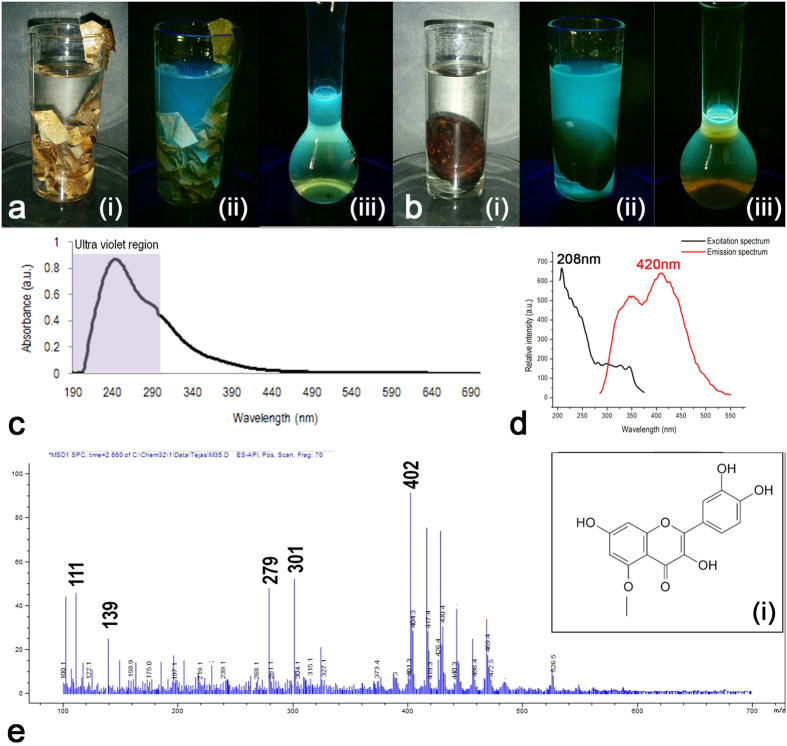
Isolation and characterization of the UV absorbing fluorophore derived from the surface of the silk cocoon and pupae. (**a**) i Photographs of tasar silk cocoon pieces immersed in methanol under white light. ii Under UV light. iii. Fluorescence of the extracted fluorophore from the silk cocoon membrane. (**b**) i Photographs of the tasar silk pupa immersed in methanol under white light. ii. Under UV light. iii Fluorescence of the extracted fluorophore from the pupal body surface. (**c**) UV-Visible absorption spectrum of the fluorophore. (**d**) Excitation and emission spectra of the isolated fluorophore. (**e**) Mass spectrum of the fluorophore, inset showing the fragmented quercetin derivative with a molecular weight of 301.

Figure 2a is showing the isolated fluorophore extracted from silk cocoon membrane. To our surprise, the same compound is also found on the body surface of the pupae (Fig. 2b). The UV absorption and fluorescent emission is shown in Fig. 2c,d; this is in agreement with our previously reported results. Figure 2e is showing the mass spectrum with a distinct peak at m/z 301, revealing presence of a quercetin derivative. It also shows, peaks at mass 111 and 402. The 402 peak is possibly due to the presence of trace amount (0.05–0.10%) of a very closely related and structurally similar flavonoid viz. nobiletin, a fungi-static flavones present in most of the plants with -OCH3 substitutions, which co-elutes along with the major flavonol fraction of quercetin in the crude extract^25^,^26^,^27^,^28^,^29^.

**Figure 3:**
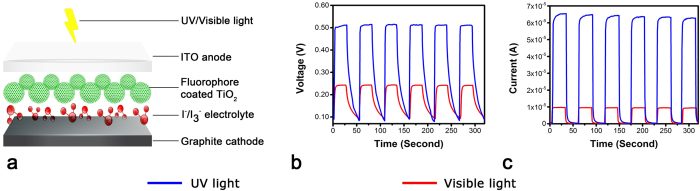
A dye sensitized solar cell device developed using the UV sensitive fluorophore derived from silk cocoon membrane and the pupae. (**a**) Overall schematic of the device. (**b**) Average voltage obtained from the device. (**c**) Average photo-current generated by the fluorophore.

The isolation of the UV sensitive fluorophore from both the cocoon and pupae, paved way to quantify the exact photoelectric effect of this compound, without any interference from the silk cocoon matrix. We developed a dye sensitized solar cell^19^,^20^,^21^,^22^ using this fluorophore as a photo-sensitizer (Fig. 3a). The details of the device are given in **methods section**. Figure 3b,c shows the voltage and current observations as a function of time. Average saturation current is 64 × 10^**−6**^ A for UV light and 10 × 10^**−6**^ A for visible light while the average saturation voltage is reported as 0.500 V for UV light and 0.230 V for visible light. In one of the earlier work, pure quercetin has been used to fabricate a quercetin/p-InP heterojunction solar cell that exhibited strong photovoltaic behavior. The observed maximum open circuit voltage (Voc) and a short-circuit current (Isc) was 0.36 V and 35.3 nA respectively, under 120 l× light intensity^30^.

## Discussion

The above results raise five interesting questions:

- What is the significance of this result in the broader perspective?
- Does the UV absorbing fluorophore play a role in strengthening the silk fiber?
- What is the origin of the potential difference across silk cocoon membrane that results in the flow of charges across the membrane?
- How does the present finding along with the previously documented electrical properties of the silk cocoon membrane influence the pupal development?
- What is the immediate impact of this study on the bio-medical interventions for human psychiatric and medical ailments?

The answers that emerge are: -

**Figure 4:**
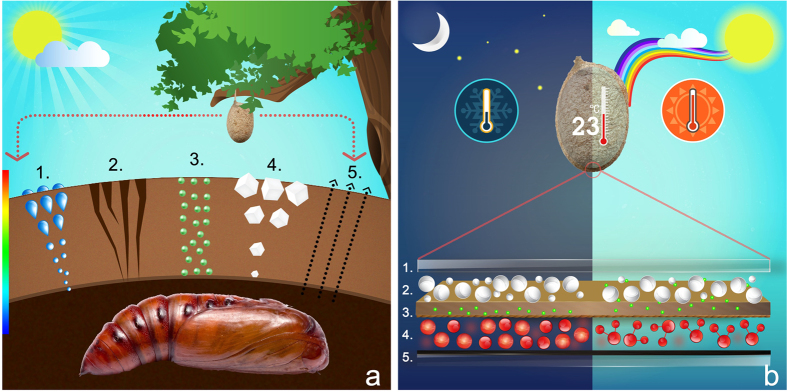
Asymmetric architecture of silk cocoon membrane and the origin of the potential difference across silk cocoon membrane. (**a**) Schematic representation of the cross section of the silk cocoon membrane showing: 1. Distribution of the water molecules in a gradient. 2. Pore sizes distribution in the silk cocoon membrane showing asymmetric tapering from outer to inner membrane. 3. Embedded fluorophore in the membrane. 4. Distribution of calcium oxalate salt crystals along with various other transition metals ions. 5. Selective carbon dioxide gating from inside to outside. (**b**) Proposed model showing a comparative mechanistic view of the silk cocoon as nature’s solar cell and comparing it with a bioanalogue, hybrid silk cocoon dye sensitized solar cell. 1. ITO Glass 2. TiO_2_ nanoparticles 3. Silk cocoon membrane 4. I^**−**^/I_3_^**−**^ electrolyte 5. Graphite electrode. On comparing with our analogous setup, high temperature outside the silk cocoon membrane served as the anode, lower temperature inside the silk cocoon served as a cathode. The fibrous matrix of the silk cocoon membrane is imparting wet semiconductor like properties. UV sensitive fluorophore is functioning as the photo sensitive dye. The trapped moisture in the silk cocoon matrix is acting as the electrolyte to improve the mobility of the charge moieties.

**Figure 5:**
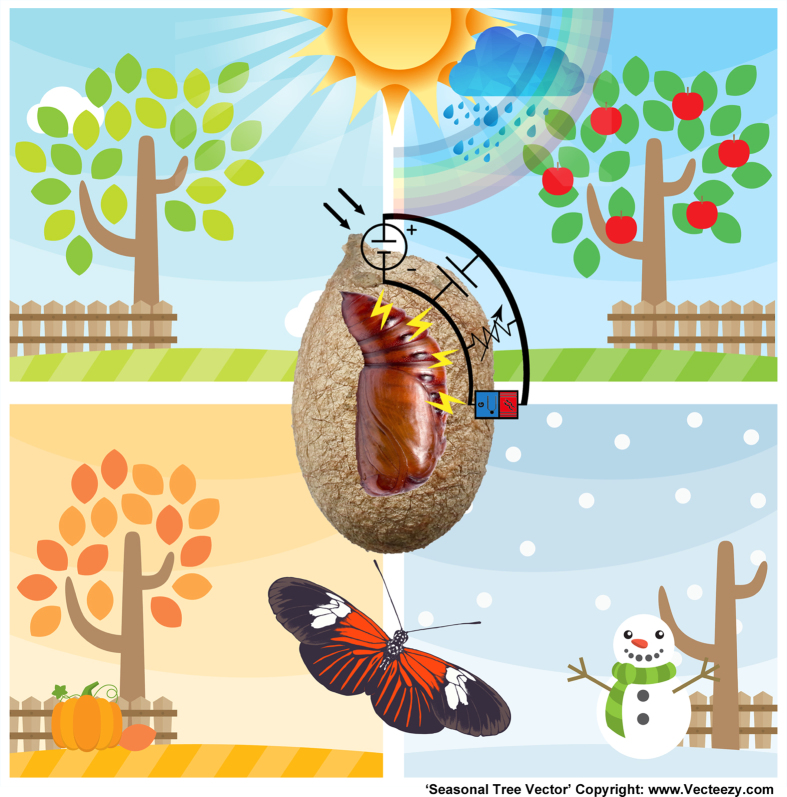
Equivalent circuit model of the silk cocoon. The overall voltage, current and resistance across the membrane changes in a diurnal and seasonal fashion. The pupae inside the cocoon possibly feels the change by being in physical contact with the inner cocoon surface. Possibly such an information transfer from silk cocoon to pupae is enhanced by the presence of moisture and salt. (Seasonal Tree Vectors- Copyright: zhaolifang and “ www.Vecteezy.com”).

- The presence of the fluorophore in the silk cocoon membrane, which all along has been believed to act as an UV shield, offers an additional functional modularity: it equips the silk cocoon as well as the pupae with the ability to convert light to electricity and especially to UV light. So while we as humans are looking for sustainable UV solar cell material, to supplement the already existing inorganic UV absorbing materials like strontium titanate^31^, here is a modest biomimetic inspiration that could be drawn from the myriad world of silkworm.
- It is already documented that UV light helps in mechanical strengthening of the spider’s silks possibly by a radical induced cross-linking reaction^32^. Has such an effect observed in wild silk cocoon of Tasar not been explored? The copious abundance of this natural UV absorbing fluorophore is pointing towards a similar role in Tasar silk.
- All these current and voltage recording are obtained by placing a synthetic anode and a cathode across the silk cocoon membrane thereby offering a directional flow of charges. How does the nature create such a vector with potential gradient across the membrane? The asymmetric structure of the silk cocoon membrane may have the key to this query. Structurally the outer surface has very high concentration of crystals of calcium oxalate salts, thus creating a salt barrier^11^. Whereas the inner surface has very little salt. Porosity of the membrane changes from the outer to inner surface, the outer being more porous than the inner surface. The inner surface is almost impermeable to water thus making silk cocoon membrane a good water proofing material^16^. The mobility of water molecules from inside to outside and vice versa is distinctly different^9^,^16^. Water molecules take less time to travel from inside to outside but takes lot more time to diffuse in the reverse direction^9^. In terms of the mobility of gas molecules, it allows carbon dioxide to move swiftly from inside to outside but the reverse flow of carbon dioxide is extremely slow thus preventing any green house kind of effect^11^. And most importantly, the whole silk cocoon has an innate ability to regulate its temperature. Earlier we have shown that irrespective of the outside temperature, a silk cocoon can maintain an ambient temperature. When we placed a cocoon at an extreme natural temperature regime of 5 and 50 °C, a temperature of 25 and 34 °C respectively was maintained inside the cocoon^11^. These asymmetric features creates a thermal, water, salt and gas gradient across the membrane and the summation of these gradients, creates the potential difference between inner and outer surface, probably being responsible for the flow of charges (Fig. 4a). It raises the possibility of developing such material in large scale for sustainable prefabricated housing material that will be able to self-regulate temperature, humidity, light and generate power, according to the changing weather conditions. In Fig. 4b, we have given a comparative picture of the silk cocoon as a natural solar cell versus the bio-analogue solar cell that we have developed using the UV sensitive fluorescent component of the silk cocoon.
- In the light of these results, we will attempt to readdress the initial question, ‘How do the brain cells of the dormant pupae senses the seasonal changes? Our current and the earlier findings indicate that the developing pupae is in physical contact with a membrane whose ‘electrical conductivity changes in a diurnal and seasonal manner. In order to visualize the changing electrical environment surrounding the pupae, we are proposing a equivalent circuit model of the silk cocoon membrane, having at least the following four circuit components viz., light to electricity converter (a nature’s solar cell), variable resistance across the membrane that changes according to the fluctuating temperature and relative humidity, thermo-electric (Seebeck) generator converting heat (depending on the temperature differences) directly into electrical energy and as a capacitor storing electrical charges. These components are connected across the silk cocoon membrane in a parallel circuit (Fig. 5). Thus at any point of time, the resultant membrane voltage and the subsequent current flow is the function of the environmental parameters and possibly a dormant pupae can sense this through charge transfer due to physical contact with the silk cocoon membrane. Similarly since the pupal body surface also has significant presence of this UV sensitive fluorophore, it also raises the possibility that upon absorbing UV light, it could generate electrical signal. Earlier it has been shown that the xanthopterin pigments present on the body shell of the oriental hornet (*Vespa orientalis*), could convert sunlight into electricity^33^. But the exact mechanism by which such electrical signals transduce into the body of the pupae or inside the body of the hornet has to be left to speculation for the time being.
- Our current understanding of this unique property of silk may have wider implication. Low cost silk mesh or silk cocoon based devices, if they retain the property of converting moisture, light, salt to electricity^9^ on surfaces like human skin, will create a revolutionary step in treatment of illnesses like chronic headache, generalized anxiety, erectile dysfunction etc. This spectrum of problems may be considered as ‘soft’ by Psychiatry but have no concrete treatment and often need long- term medication. If silk based mesh is able to utilize the moisture and salt from the human skin and convert it to a micro current it can serve as a personalized biofeedback mechanism and relaxation device and an alternative to transcranial direct current devices that are being tested to ameliorate depression and anxiety. These would be essentially low cost due to abundance of silk. Similarly low intensity electric shocks have been shown to improve erectile dysfunction not otherwise responsive to medication at least in one experiment in Israel^34^,^35^,^36^,^37^. Further experimentation is being done by our team to discover whether ‘Silk’ in contact with skin moisture and salt in pubic area or skin can generate this current as it does on the surface and possibly stimulate the penile blood flow, bypassing the chemical triggering.

## Methods

### Source of the tasar silk cocoon

Silk cocoons of silk moth *Antheraea mylitta* (Tasar) were used for this study. Cocoons were procured from Jharcraft, a commercial tasar silk industry situated in the city of Ranchi in Jharkhand state of India. These were live cocoons viz. they had a dormant pupa residing inside each one of them. Cocoons were stored in a low light chamber with enough ventilation for gas exchange. These were further used for this study, by carefully cutting the silk cocoon, and thereafter removing the pupae.

### Raw silk cocoon solar cell device

A graphite electrode (φ = 2 mm), cathode, was inserted from one end (1) of an empty cocoon shell ensuring that it touches the other end (2) from inside. A conducting, indium tin oxide coated glass (ITO), anode, was placed from outside of the end (2) with conducting surface touching the cocoon shell as explained in Fig. 1a. This setup was moistened for 20 seconds in order to allow water molecules to fill the pores. The setup was exposed to light from end (2) in a Faraday’s cage. ITO glass as anode and a graphite rod as the cathode is chosen for the raw silk cocoon solar cell device because Indium-tin oxides (ITO) is one of the most widely used materials as front contact transparent conducting oxides for hetero-junction solar cells application. This is primarily because of the high optical transmittance of ITO in the visible region, surface uniformity, high electrical conductivity, and industrial process compatibility. The limited availability of indium on earth’s crust makes, ITO highly expansive. So as to reduce the cost, researchers are currently working on other transparent conducting materials such as PEDOT:PSS. These materials have shwn improved performance comparable to the corresponding devices made from traditional ITO anode. Generally a catalytic material like platinum or carbon is used as the positive terminal of the cell is a catalytic material such as platinum or carbon, so as to catalyze the electron transfer process and for the circuit to be complete. So we used graphite is used as cathode^38^,^39^,^40^,^41^. In one of our earlier studies we have also tested platinum and other electrode materials and found the recordings to be comparable between different electrodes^42^.

### Hybrid silk cocoon DSSC

TiO_2_ paste was made in water by mixing TiO_2_, Triton X100 in concentration of 10%w/v, 0.3% v/v respectively. Ethylene glycol (20% v/v) in I_2_-KI solution (0.5 M) was employed as electrolyte. Indium tin oxide coated glass (ITO) was properly cleaned by sonication in distilled water (DW) and ethanol. Conducting side of the ITO (2 cm × 2 cm) was uniformly spray coated with TiO_2_ paste. TiO2 coated ITO was heated by gradually increasing temperature from 200 °C to 500 °C for four hours and used as an anode after cooling. Silk cocoon pieces (1 cm × 1 cm) were washed in DW. The wet pieces were pressed in between glass slides ensuring that they maintain firm contact and were dried in hot air oven at 50 °C. I_2_-KI electrolyte (25 μl) was uniformly spread on dried silk cocoon piece. Graphite sheet (1.8 cm × 1.8 cm × 2 mm) was used as a cathode. Sandwiching I2-KI coated cocoon piece in between anode and cathode made the final ‘hybrid silk cocoon DSSC’. Additional few drops of electrolyte were added on the edges, ensuring a uniform spread throughout the device. (Refer to supplementary information for additional precautions while preparing the solar cell).

### Extraction of fluorophore

Fluorophore from both, tasar silk cocoon and pupa was extracted separately using methanol. Silk cocoons were cut into small pieces and immersed in methanol for 24 hours with continuous stirring. Similarly, the pupae were immersed in methanol for 24 hours. Both cocoon pieces and pupae were mixed in separate methanol solution, in the ratio of 10% w/v. The mixtures were kept at 25 °C. Later the mixtures were filtered using Whatman’s filter paper. In order to concentrate the filtrate, it was kept on a heating plate at 70 °C until a dark brownish color was obtained. This concentrated filtrate was further used for making DSSC.

The color of the pupal methanol extract is yellowish brown. It becomes dark brown as it gets concentrated during evaporation. We checked the stability of the fluorophore by measuring fluorescence at different temperatures. To our surprise, the fluorescence intensity of the fluorophore was intact even at 80 °C (Fig. S1, supplementary information). Considering the boiling point of methanol, we selected 70 °C for evaporation^10^,^23^,^24^.

### UV-Vis spectroscopy

Spectrum of diluted fluorophore in methanol was measured using LabIndia UV-VIS 3000 spectrophotometer.

### Fluorescence spectroscopy

Fluorescence excitation and emission spectra were measured using Perkin Elmer L5 55 fluorescence spectrophotometer with slit size 7.

### Mass spectrometry

Spectrum was obtained using Agilent Technologies 1260 Infinity and 6120 Quadrupole LCMS using Methanol as a solvent and operating at a positive mode.

### Fabrication of the DSSC cell

TiO_2_ coated ITO, anode, was immersed in the fluorophore for 3 hours and later dried at 50 °C with 25 μl of electrolyte uniformly spread onto it. Final device was made using cathode as explained previously in methods. Additional electrolyte was added from the edge to achieve its uniform distribution in the device.

## Additional Information

**How to cite this article**: Tulachan, B. *et al.* The role of photo-electric properties of silk cocoon membrane in pupal metamorphosis: A natural solar cell. *Sci. Rep.* **6**, 21915; doi: 10.1038/srep21915 (2016).

## Supplementary Material

Supplementary Information
