# Supplementary material for: The role of photo-electric properties of silk cocoon membrane in pupal metamorphosis: A natural solar cell
Source: Sci Rep. 2016 Feb 24;6:21915. doi: 10.1038/srep21915 (PMC4764832; doi:10.1038/srep21915)
Supplement: Supplementary Information [file srep21915-s1.pdf]

Supplementary information

The role of photo-electric properties of silk cocoon membrane in pupal metamorphosis: A natural solar cell

Brindan Tulachan[1], Shivansh Srivastava[2], Tejas Sanjeev Kusurkar[1], Niroj Kumar Sethy[3], Kalpana Bhargava[3], Sushil Kumar Singh[4], Deepu Philip[5,6], Alok Bajpai[7], Mainak Das[1,6]*

[1]Biological Sciences & Bioengineering, Indian Institute of Technology Kanpur, Kanpur, Uttar Pradesh, 208016, India

[2]Department of Chemical Engineering, Delhi Technological University, Shahbad Daulatpur, Main Bawana Road, Delhi, 110042, India

[3]Defense Institute of Physiology & Allied Sciences, Defense Research Development Organization, Timarpur, Lucknow Road, Delhi, 110054, India

[4]Solid State Physics Laboratory, Defense Research Development Organization, Timarpur, Lucknow Road, Delhi, 110054, India

[5]Industrial & Management Engineering, Indian Institute of Technology Kanpur, Kanpur, Uttar Pradesh, 208016, India

[6]Design Program, Indian Institute of Technology Kanpur, Kanpur, Uttar Pradesh, 208016, India

[7]Institute Psychiatrist, Indian Institute of Technology Kanpur, Kanpur, Uttar Pradesh, 208016, India

Corresponding author: Mainak Das, email: mainakd@iitk.ac.in

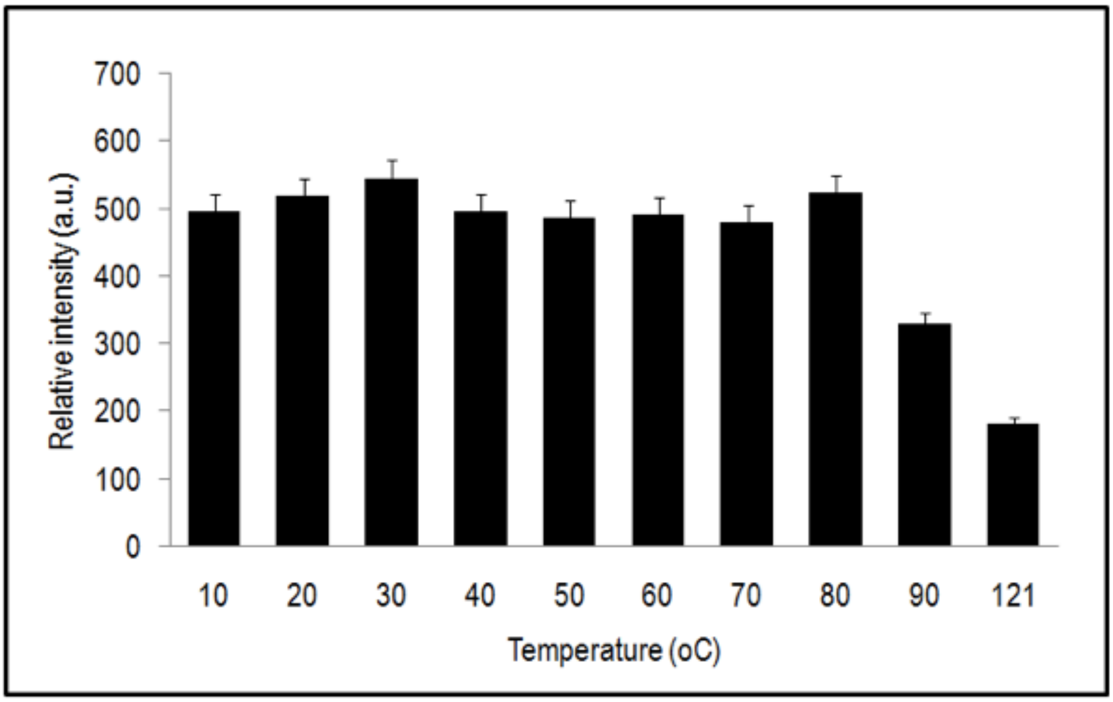

Figure S1. Effect of different temperatures on the fluorescence intensity of crude fluorophore extract obtained from Tasr silk cocoon membrane.

Precautions to be taken to spread the electrolyte uniformly on the cocoon surface:

Even after spreading electrolyte uniformly, during sandwiching the electrodes there would be small pockets of air within the cell. As we know silk cocoon membrane is very porous in nature and those electrolyte would get soaked in and create pockets of air. This is the reason we added drops of electrolyte afterwards.
